# Extracts and Marine Algae Polysaccharides in Therapy and Prevention of Inflammatory Diseases of the Intestine

**DOI:** 10.3390/md18060289

**Published:** 2020-05-31

**Authors:** Natalya N. Besednova, Tatyana S. Zaporozhets, Tatyana A. Kuznetsova, Ilona D. Makarenkova, Sergey P. Kryzhanovsky, Lydmila N. Fedyanina, Svetlana P. Ermakova

**Affiliations:** 1 Somov Institute of Epidemiology and Microbiology, Vladivostok 690087, Russia; besednoff_lev@mail.ru (N.N.B.); takuznets@mail.ru (T.A.K.); ilona_m@mail.ru (I.D.M.); 2 School of Biomedicine, Far Eastern Federal University, Vladivostok 690087, Russia; kryzhanovskii.sp@dvfu.ru (S.P.K.); fedyanina.ln@dvfu.ru (L.N.F.); 3 G.B. Elyakov Pacific Institute of Bioorganic Chemistry, FEB RAS, Vladivostok 690022, Russia; swetlana_e@mail.ru

**Keywords:** inflammatory bowel disease, Crohn’s disease, ulcerative colitis, marine algae polysaccharides

## Abstract

Inflammatory bowel disease (IBD) is a serious public health problem worldwide. Current therapeutic strategies that use anti-inflammatory drugs, immunosuppressants, and biological treatments are often ineffective and have adverse health effects. In this regard, the use of natural compounds aimed at key pathogenic therapeutic targets in IBD attracts universal attention. Seaweed is a valuable source of structurally diverse biologically active compounds. The materials presented in the review indicate that seaweed extracts and polysaccharides are effective candidates for the development of drugs, biological food additives, and functional nutrition products for the treatment and prevention of IBD. The structural features of algal polysaccharides provide the possibility of exposure to therapeutic targets of IBD, including proinflammatory cytokines, chemokines, adhesion molecules, nuclear factor NF-kB, intestinal epithelial cells, reactive oxygen and nitrogen. Further study of the relationship between the effect of polysaccharides from different types of algae, with different structure and molecular weights on immune and epithelial cells, intestinal microorganisms will contribute to a deeper understanding of their mechanisms and will help in the development of drugs, dietary supplements, functional foods for the treatment of patients with IBD.

## 1. Introduction

Inflammatory bowel disease (IBD) is a group of idiopathic chronic inflammatory intestinal conditions. The two main disease categories are Crohn’s disease (CrD) and ulcerative colitis (UC), which occupy one of the leading positions of diseases of the gastrointestinal tract due to the severity, frequency of complications and mortality [1,2]. Late diagnosis and inadequate treatment lead to the development of complications, including the occurrence of malignant growth, as well as disabled people of working age and death. Since the middle of the 20th century, the incidence of IBD has been increasing everywhere, is on average 4.1 per 100,000 population for UC and 0.8 per 100,000 population for CrD [2,3,4,5,6]. The prevalence of IBD has also increased significantly, turning these diseases into a public health problem worldwide [7].

The etiology of IBD is still unknown, despite a long history of study. From a modern perspective, the pathogenesis of IBD is considered as the interaction of several factors Advances in gene sequencing technology have identified more than 160 susceptibility genes associated with IBD, but these genes are not the main reason for the increase in the number of diseases [8,9]. The rapid rise of IBD incidence in certain geographic regions suggests that disturbance of the immune system and/or imbalanced interactions with microbes leads to the development of chronic intestinal inflammation when certain environmental factors, including exposure to drugs (such as antibiotics), viruses, psychological stress, air pollutants, diet, and chemicals contribute to trigger genetically susceptible hosts [10,11,12]. 

Immunological dysregulation in IBD is characterized by dysfunction of the effector and regulatory cells of innate acquired immunity, which leads to the uncontrolled release of soluble inflammatory mediators with the subsequent development of pathological changes [13,14]. T cells are activated in both diseases, but the level of differentiation and activation of T-cell immunity is different in CrD and UC. CrD is usually designated as a Th1 and Th17 condition with elevated production of IL-12, IL-23, IFN-γ, and IL-17, whereas UC is usually characterized as a Th2 condition with increased production of IL-13, IL-5, and IL-9 [13,15,16]. It is also well documented that downregulation of IL-10 promotes disease progression in patients with IBD [15,17]. On the other hand, a lot of new information accumulated on a defective innate immunity as the primary mechanism involved with the development of IBD [18]. Innate immunity provides an answer through recognition of pathogen-associated molecular structures (PAMP), which include Nod-like-receptor (NLRs), mannose receptors, scavenger-receptor and Toll-like receptors (TLRs). Abnormal regulation of these signaling pathways during both the early and chronic phases of intestinal inflammation may result in a persistent inflammatory process, which may underlie the pathogenesis of IBD [19]. Finally, the development of new molecular genetic tools made it possible to study in more detail the key mechanisms of the effect of microbiota on the intestines in IBD [20,21].

According to the current recommendations, anti-inflammatory drugs (5-aminosalicylic acid derivatives, corticosteroids) and immunosuppressants (thiopurines, antimetabolites, calcineurin inhibitors), biological drugs (anticytokine drugs, adhesion molecule antagonists, antibiotics) can be used for the treatment of IBD [22], surgical procedures to repair or remove affected areas of the gastrointestinal tract, as well as non-pharmacological alternative methods, can be used to treat IBD [23]. However, their use is often not safe for the body and is associated with side effects. Aminosalicylates cause headaches and nausea. Corticosteroids can lead to the development of osteoporosis, hypertension, obesity, type 2 diabetes, exacerbation of gastrointestinal ulcers. Anti-TNFα drugs can cause allergic and anaphylactoid reactions, infectious complications, cardiological symptoms, skin lesions. Moreover, TNF inhibitors are not useful for one-third of all patients (i.e., <primary failures>), and a further one third lose effect over time (<secondary failures>) [24].

Given the serious side effects associated with conventional treatment, the search for new sources of drugs to improve the clinical symptoms of IBD remains essential. Recent advances in the study of IBD pathogenesis have opened up prospects for the development of new treatment strategies aimed at therapeutic targets and associated with the use of areactogenic and effective natural compounds that can block the adhesion and migration of leukocytes into the inflamed intestinal mucosa and inhibit cytokines, normalize the intestinal microflora, maintain clinical remission and accelerate mucous healing intestinal membrane [25,26].

In this regard, marine algae represent a huge bioresource of natural ingredients [27,28]. Recently, studies have been actively developed related to the possibility of using complex glycans (polysaccharides) as therapeutic agents [29,30,31,32]. A wide range of biological activity of polysaccharides―immunomodulating, antitumor, antiviral, antioxidant, lipid-lowering―is closely related to their monosaccharide composition, molecular weights, types of bonds and chain conformation [33] and is reflected in the reports of both preclinical and clinical trials [33,34]. A large number of experimental data indicates that algal polysaccharides, as well as algae extracts, have an anti-inflammatory and gastroprotective effect and are very promising for the treatment and prevention of gastrointestinal diseases [35,36,37,38,39]. Algae polysaccharides are resistant to the action of gastric juice, the digestive enzymes of the host, are not absorbed in the upper sections of the gastrointestinal tract, and can serve as fermentation substrates for specific intestinal microbial populations [40,41]. Marine polysaccharides also have great potential for drug delivery systems [30]. 

This work includes a review of current ideas about the possibility of using extracts and polysaccharides of seaweed as the basis for drugs, dietary supplements and functional food products for the prevention and treatment of inflammatory bowel diseases.

## 2. Algae Polysaccharides

### 2.1. Brown Algae Polysaccharides

Brown seaweed is a large group of multicellular photoautotrophic organisms. Alginates and fucoidans are two of the best-known seaweed biopolymers and have experimentally and clinically proven pharmacological effects, such as hypolipidemic, hypoglycemic, antioxidant, anti-inflammatory, anticoagulant, metal-binding, and immunomodulating [32,35,37,42,43,44].

Fucoidans are complex highly sulfated, usually branched polysaccharides in the range from several tens to several thousand kDa, often containing, in addition to fucose residues, glucose, galactose, xylose, mannose and uronic acids, as well as acetyl groups [44,45]. Fucoidans are present mainly in the matrix of the cell wall of various types of brown algae and also perform important structural and protective [45,46]. The potential application of fucoidan in pharmaceutical technology is also due to its ionic nature. The negative charge of the molecule results from the presence of sulfate residues in the C-2 and C-4 positions, occasionally in C-3, allowing the formation of complexes with other oppositely charged molecules [47]. Fucoidan has a low-toxicity, is biodegradable [30] and is a biocompatible compound approved by the Food and Drug Administration (FDA) in the Generally Recognized as Safe (GRAS) category as a food ingredient [47]. Fucoidans might exhibit antioxidant [43], anticoagulant [48], antibacterial, antifungal [49], anti-inflammatory, and immunomodulatory [50] activity.

Alginic acids are found in all brown seaweeds (Phaeophyceae) in a proportion of 18% to 40% of the total plant mass [46]. These anionic polysaccharides belong to the family of linear copolymers and consist of residues of β-d-mannuronic and α-l-guluronic acids [51]. Alginates (water-soluble salts of alginic acid) are biocompatible, non-toxic, non-immunogenic and biodegradable [46]. The alginates are known to form a physical gel by hydrogen bonding at low pH (acid gel) and by ionic interactions with divalent or trivalent ions, which act as crosslinkers between adjacent polymer chains [46]. Alginate hydrogels are widely used as biomaterials [52]. Alginic acid also plays an important role as fiber for maintaining the health of animals and humans [27].

Laminarans are low molecular weight (5–10 kDa) β-d-glucans which consist of β-(1→3)-linked d-glucose residues. They differ from each other with regard to their length and branching structure [53,54]. The content of laminaran in some types of brown algae is from 10% to 35% of the dry weight [32]. Laminarans possess diverse biological properties—antioxidant, antitumor, antimicrobial, immunomodulating, prebiotic and anticoagulant [55,56,57]. 

### 2.2. Red Algae Polysaccharides

The main structural component of red algae is sulfated polysaccharides (galactans)—carrageenans, agars, porphyrin, xylan—which have no analogs among other plant polysaccharides. The structural features of these polysaccharides are determined by the species affiliation of algae, their habitat conditions and determine a wide range of biological activity [32].

Carrageenans are a family of sulfated hydrocolloids whose polymer chain is built from galactose residues and its derivatives with alternating α-(1-3) and β-(1-4) bonds. Carrageenans are classified by location and the amount of sulfate groups in the monosaccharide residues and the presence of 3,6-angirogalactose in 4-*O*-substituted residues and are divided into carrageenans kappa (k), iota (i) and lambda (λ) [32,58]. Carrageenans are the most studied among algae polysaccharides in terms of toxicity, pyrogenicity and allergenicity and food safety and medical use [59,60]. Carrageenans have immunomodulating, antioxidant, antitoxic, antiviral, antitumor and anticoagulant properties [61]. Recently, the utility of carrageenans as gelling- or viscosity-enhancing agents for controlled drug release systems [60]. However, taking into account three differences in the properties of the most important types of carrageenans (κ, τ, λ), the safety assessment should be very thorough.

### 2.3. Green Alga Polysaccharides

Ulvan is the major water-soluble polysaccharide found in green seaweed of the order Ulvales (*Ulva* and *Enteromorpha sp.*) that has sulfate, rhamnose, xylose, iduronic and glucuronic acids as the main constituents [58,62]. The structure of the disaccharide moieties of ulvans resembles that of glycosaminoglycans, which occur in the extracellular matrix of connective tissues of animals [32]. Ulvan and its oligosaccharides have various biological effects, such as anticoagulant, antiviral, antiallergic, antitumor, anti-inflammatory etc. [62].

## 3. Experimental Models of IBD

Success in understanding the intestinal inflammation that occurs with inflammatory bowel disease has been achieved through the development of mouse models of intestinal inflammation [63]. There are two main approaches to modeling UC and CrD [64]. The first of these consists in obtaining spontaneously developing diseases in mice with a certain genotype or as a result of exposure to the genome (knockout or transgenic mice), which is associated with a loss of immune tolerance to autogenic intestinal microflora. The second approach is to simulate chemically induced colitis. For this, trinitrobenzenesulfonic acid (TNBS), sodium dextran sulfate (DSS), oxazolone (4-ethoxymethylene-2-phenyl-2-oxazolin-5) and others are used. TNBS elicits a Th-1-dependent immune response and oxazolone-Th-2 phenotype. Intrarectal administration of the TNBS renders colonic proteins immunogenic to the host immune system and thereby initiates a mucosal immune response that drives colitis in susceptible mouse strains [65]. Administration of oxazolone elicits colonic inflammation that differs markedly from that caused by TNBS and that resembles many features of ulcerative colitis rather than CrD [66].

As some of these characteristics resemble features of CrD, TNBS colitis has been widely used in the study of immunologic aspects relevant to this disease, including cytokine secretion patterns, mechanisms of oral tolerance, and effects of potential immunotherapies. However, experimental colitis in mice is most often modeled using DSS, which mice receive with drinking water. This model is popular due to the ease of reproduction and the speed of obtaining the effect, depending on the time of exposure and dose [67]. In this case, mice with DSS-induced colitis exhibit phenotypic characteristics similar to acute and chronic colitis in humans (rectal bleeding, weight loss, diarrhea, ulceration of the submucous membrane, violation of the structure of crypt cells, penetration of a large number of cells into the colon mucosa). Macroscopic and histopathological changes are associated with excessive production of pro-inflammatory cytokines [68]. Importantly, this inflammation develops in the absence of T cells mediating adaptive immunity, that effector cytokines produced by innate cells are sufficient to cause the inflammation. On this basis, DSS colitis has become a useful model for the study of the innate immune mechanisms involved in the development of intestinal inflammation [64].

In vitro cell culture models provide a useful tool for studying IBD. Immortalized cell lines of animal and human origin, including IEC Caco-2, IPEC-1, and IPEC-J2, are used to study the effects of drugs and food elements on the barrier function of intestinal epithelium, paracellular permeability, cytokine production and mucosal immune response in IBD [69,70]. The advantages of using IPEC-J2 as an in vitro model of the GI tract are the high resemblance between humans and pigs, and the ease of extrapolating in vitro to in vivo characteristics [69]. Human Caco-2 cells originally isolated from a human colon adenocarcinoma, after 18–21 days of culture become a homogenously polarized monolayer of enterocyte-like cells with apical and basolateral membranes, a brush border with microvilli and TJ [71]. Caco-2 cells express TLR and produce various cytokines [72], which allows them to be used to evaluate substances with both immunomodulating properties and the ability to protect damaged intestinal epithelium [73,74,75]. The cell transfer model used with great effect to define the role of regulatory cells in intestinal inflammation and establish the fact that mucosal homeostasis depends on a balance between mucosal proinflammatory effector function and anti-inflammatory regulatory function [76].

## 4. Therapeutic Targets for Algae Polysaccharides in IBD

### 4.1. Targeting Pro-inflammatory Pathways

#### 4.1.1. Pro-inflammatory Cytokines

It has now been clearly established that cytokine responses are key elements that control the inflammatory mechanisms that underlie IBD [13,16,25,77]. Cytokines not only drive intestinal inflammation and diarrhea in IBD but may also regulate extra-intestinal disease manifestations (for example, arthralgia or arthritis) and systemic effects. Furthermore, cytokines seem to have a crucial role in driving complications of IBD such as intestinal stenosis, fistula formation and colitis-associated neoplasias [77]. Studies using tissue from patients with IBD and animal models of IBD have identified cytokine spectrum as potential new targets for the therapy of intestinal inflammation [76]. Such targets include IL-17, the main pathogenetic factor IBD, which stimulates a strong chronic immune-inflammatory response [78], pro-inflammatory cytokines IL-6, IL-12, IL-23 and IL-21, as well as anti-inflammatory cytokines, such as IL -10 and TGF-β [16,77]. Theoretically, potential strategies would include the blockade of Th17 cell differentiation and expansion, the neutralization of the cytokines produced by these cells, and the inhibition of the specific transcription factors required for Th17 cell function [79,80,81]. The key role of cytokines is also highlighted by the fact that blockade of TNF is now commonly used as a standard therapy for IBD in the clinic [82].

Brown algae polysaccharides are promising candidates as a therapeutic alternative for patients with IBD due to the fact that these compounds modulate many pro-inflammatory mechanisms and mediators including regulation of gene expression of pro- and anti-inflammatory cytokines related to ulcerative colitis [32,42,50,83,84].

Matsumoto et al. [85] examined the preventative of various types of fucoidans on the production of IL-6 in the line of colon epithelial cells CMT-93 stimulated by lipopolysaccharides in vitro and in vivo effect on chronic colitis of mice induced by sodium dextran. Fucoidans from *Cladosiphon okamuranus Tokida* and *Kjellmaniella crassifolia* have been shown to inhibit IL-6 production in CMT-93 cells and decrease NF-kB nuclear translocation. Fucoidan from *Cladosiphon okamuranus* also inhibited the synthesis of IFNγ and IL-6 and increased the synthesis of IL-10 and TGF-β in the colon lamina propria reduced the expression level of IL-6 mRNA in mouse epithelial cells compared to mice fed a standard diet. Disease activity index and myeloperoxidase activity also decreased in mice treated with *Cladosiphon okamuranus* fucoidan. Ryan et al. [86] showed that β-glucan obtained from *Laminaria hyperborea and Laminaria digitata* can both significantly decrease the expression of Th17-associated cytokines (IL-17a, IL-17F, and IL-22) as well as receptor IL-23R and IL-6, with no alteration to the T regulatory cell (TREG)–related targets.

O’Shea et al. [87] studied the impact of prior consumption of laminaran and/or and fucoidan on pathology and inflammation following DSS challenge in pigs. The findings of this study show that prior exposure to diets containing and fucoidan and a combination of and fucoidan and laminaran together, ameliorated weight loss, diarrhea, but failed to improve the pathology score associated with a DSS challenge in the proximal colon of pigs. Pigs receiving both LAM and/or FUC prior to the onset of a DSS challenge had decreased IL-6 mRNA abundance.

Lean et al. [83] evaluated the therapeutic potential of fucoidan-polyphenol complex (Maritech**^®^** Synergy, which is a highly characterized, certified organic complex of fucoidan and marine polyphenols, sourced from wild *Fucus vesiculosus* seaweed) and depyrogenated fucoidan in DSS mouse model of acute colitis and depyrogenated fucoidan in DSS mouse model of acute colitis. Orally administered polysaccharides significantly ameliorated symptoms of colitis based on retention of body weight, as well as reduced diarrhea and fecal blood loss, compared to the untreated colitis group. Colon and spleen weight in mice treated with oral fucoidan was also significantly lower, indicating reduced inflammation and edema. The macroscopic changes induced by oral fucoidan correlated significantly with substantially decreased production of inflammatory cytokines by the colon tissue. It is noteworthy that deterioration in the condition of animals and an increase in the level of certain pro-inflammatory cytokines in the colon tissue was noted with intraperitoneal administration of depyrogenized fucoidan. The authors propose the oral use of fucoidan as an effective and well-tolerated maintenance therapy for a long period of time to reduce inflammation and maintain the integrity of the intestinal epithelium.

Tanoue et al. [73] in an in vitro model for co-culture of intestinal epithelial cells of Caco-2 and macrophage cells RAW264.7 have established that fucoidan inhibits the expression of the IL-8 gene in epithelial cells by reducing the production of TNF-α by macrophages stimulated by lipopolysaccharide.

The authors of these publications suggest that algae polysaccharides could, therefore, represent a novel nutraceutical option for the management of IBD and suggest using them as an effective and well-tolerated maintenance therapy for a long period of time to reduce inflammation and maintain the integrity of the intestinal epithelium.

Treatment with the methanolic extract significantly attenuates body weight loss and severe clinical symptoms in mice with experimental colitis induced by DSS. This was associated with a remarkable amelioration of colonic architecture and a significant reduction in pro-inflammatory cytokine production in the intestinal tissue. The authors attribute this effect to the ability of the extract to reduce the rate of migration of lymphoid cells into the focus of inflammation and directly inhibit the secretion of cytokines by immunocytes [88,89].

The authors also believe that these effects on clinical symptoms and on histological parameters could be due to the presence of antioxidant compounds, such as β-carotene and α-tocopherol that have been isolated from *Caulerpa mexicana* [90] and inhibition of the formation of free radicals and the suppression of oxidative stress can be one of the important factors causing a decrease in the intensity of damage to the intestinal epithelium [91].

The efficacy of oral administration of an ethanol extract of the red alga *Eucheuma cottonii* with a high content of polysaccharides in experimental colitis is described by Sudirman et al. [5]. Extract administration protected against weight loss and decreased the colon weight per length ratio. The intestinal mucosa of the control mice was thickened and eroded, while the mucus morphology was preserved in the animals treated with the extract. The level of pro-inflammatory cytokines (TNFα, IL-1β and IL-6) in the blood serum, as well as IL-1β in the colon tissue of mice with colitis receiving algae extract, was lower than that of control animals (pronounced manifestations of colitis). The level of pro-inflammatory cytokines (TNFα, IL-1β and IL-6) in the blood serum, as well as IL-1β in the colon tissue of mice with colitis receiving algae extract, was lower than that in control animals, and the level of anti-inflammatory interleukin IL-10 was higher.

#### 4.1.2. Intercellular Adhesion Molecules

Lymphocyte-endothelial interactions mediated by adhesion molecules, such as selectins, integrins, cadherins, etc., play an important role in the migration and involvement of leukocytes in places of inflammation and can also be used as potential therapeutic targets for chronic intestinal inflammation [92]. Selectins that bind to the mannose and fucose terminal residues of polysaccharides and glycoconjugates slow rolling of leukocytes and platelets to the surface of the endothelium of the vessel walls, facilitating transendothelial transmission [93]. The spatial pattern of sulfated saccharide structures of fucoidan, that imitates the clustering of sulfated, sialylated, and fucosylated oligosaccharides on the cell surface, provides binding to L- or P-selectins [94]. The interaction between fucoidan and selectins has physiological consequences that may be therapeutically beneficial. So, Semenov et al. [95] observed a significant suppression of the release of neutrophils into the abdominal cavity upon intravenous administration of fucoidan for 15 min–1.5 h after intraperitoneal administration of peptone to rats with experimental peritonitis. According to the authors, the blockade of inflammation at an early stage of its development occurs due to the interaction of fucoidan with P-selectin.

Zhang et al. [96] also found that intravenous administration of fucoidan reduced colonic mucosal damage and crypt destruction of dextran sodium sulfate-induced murine chronic colitis by reducing abolishing venular leukocyte rolling and extravascular recruitment. Most likely, fucoidan is acting like heparin or heparan sulfate (HS), presenting a spatial pattern of sulfated saccharide structures that imitates the clustering of sulfated, sialylated, and fucosylated oligosaccharides on the cell surface. Fucoidan is also able to block β2-integrin-dependent adhesion of leukocytes on intestinal epithelial cells by binding to CD11b/CD18 [97].

These results prove that sulfated polysaccharides, possessing the properties of glycosaminoglycan mimetics, block adhesion molecules and inhibit the migration of leukocytes, having a positive effect in the treatment of inflammatory bowel diseases

#### 4.1.3. Active Oxygen and Nitrogen Species

The data accumulated to date show that oxidative stress plays a significant role in the pathogenesis and progression of IBD, being a potential pathogenic and critical factor in the occurrence, progression, and severity of the disease, and not a consequence of chronic inflammation of the intestinal mucosa [98,99]. Several literature reports have shown an increase of active forms of oxygen and nitrogen in patients with IBD [99,100] as well as in models of mouse colitis [101]. Defective antioxidant protection is another key factor contributing to the progression of IBD [99]. In this regard, potential therapeutic strategies for IBD aimed at signaling oxidative stress and involving the use of natural and synthetic antioxidant compounds are reviewed and discussed. Polysaccharides and algae extracts have pronounced antioxidant properties [38,44,102,103,104,105] and are potential therapeutic substances that target oxidative stress in IBD. However, further studies are needed to confirm the exact effects of these promising drugs and the suitable doses and administration routes.

### 4.2. Other Therapies

#### 4.2.1. Intestinal Epithelial Cells (IEC)

An important role in the pathogenesis of IBD is given to reducing the effectiveness of the epithelial barrier. The epithelial barrier consists of several important elements, including the intact epithelial monolayer and the locking intercellular contacts (tight junctions, TJ) in which the membranes of neighboring cells are maximally brought together and are “cross-linked” by specialized proteins claudins and occludins [105]. A large body of evidence suggests that the disruption of the ECCs, TJ abnormalities and increased paracellular permeability contributes to excessive antigenic stimulation, which leads to the development of an immune-mediated inflammatory response in the intestinal wall and, ultimately, to IBD [105,106,107,108]. In this regard, the restoration of intestinal barrier function might be effective in preventing disease in at-risk individuals or maintaining remission in patients with IBD [109].

Iraha et al. [110] showed that fucoidan prevented H_2_O_2_-induced destruction of intestinal epithelial barrier function in a dose-dependent manner significantly increased transepithelial resistance, which indicates a direct increase in the barrier function of the intestinal epithelium and prevented a violation of paracellular permeability, increasing the expression of claudin-1. The authors suggest that fucoidan, which increases the epithelial protective function and promotes epithelial regeneration, might serve as an appropriate therapy for the treatment of IBD.

Yang et al. [111] investigated the potential anti-inflammatory and protective properties of intestinal permeability of an aqueous extract of *Laminaria japonica* and three types of fermented aqueous extracts of *L. japonica* in LPS-stimulated Caco-2 cells. All four extracts enhanced intestinal barrier function, preventing inhibition of tight junction-related protein levels, decreased monolayer permeability, and significantly reduced nitric oxide and IL-6 production.

A significant increase in the intestinal epithelial barrier and the immune function of cells when using low-molecular-weight fucoidan and high-stability fucoxanthin was shown in the same model [112].

Thus, the above examples show that algae polysaccharides can affect intestinal health by decreasing intestinal inflammation and enhancing barrier function by partially regulating dense compounds and associated proteins (claudine, occludine). Further studies of more TJ-linked proteins will establish the exact mechanism of action of polysaccharides and confirm their potential benefit as a suitable therapeutic agent for the treatment of inflammatory bowel diseases.

#### 4.2.2. Microbiota

The concept of altered intestinal microbiome or dysbiosis is the significant IBD research event of the last decade. Newer molecular and genetic diagnostic tools have elucidated distinct changes in the gut microbiota in IBD patients and clarified the deficiencies of innate immunity [112]. IBD began to be seen as an immunodeficiency state, and impaired microbiome as a key factor in the long course of inflammation [113,114]. In this regard, manipulations of the intestinal microbiome, including the use of antibiotics, prebiotics, probiotics and fecal microbiota, are an attractive alternative for both prophylactic and therapeutic intervention in IBD [20,115,116,117].

According to the latest definition, prebiotics include substrates that are selectively used by host microorganisms that provide health benefits [118]. Substances claiming to be used as a prebiotic must meet three main criteria: be resistant to absorption and the action of enzymes in the upper gastrointestinal tract, must be fermented by colon microflora, and also be a selective substrate for the growth of beneficial bacteria and cause local or systemic effects beneficial to the health of the host [118]. Mono- and oligosaccharides meet the specified criteria to the greatest extent, although, in theory, any dietary material that is not absorbed by entering the colon can potentially be considered a prebiotic. Experimental [119] and clinical studies [120] have shown that prebiotics provide a favorable environment for the growth of probiotic strains in the intestine, inhibit potentially pathogenic bacteria, reduce inflammation of the mucous membrane, and reduce the risk of subsequent clinical relapses of IBD [116,119,120,121,122].

In recent years, much attention has been paid to the use of polysaccharides from algae as prebiotics, which are not broken down by upper enzymes and can be degraded in the colon by enzymes of bifidobacteria and lactobacilli, such as xylanases and glycosyl hydrolases, phosphate transferases, hydrolases and isomerases [34,40,41,123,124,125]. Algal polysaccharides and oligosaccharides have advantages over other sources for the production of prebiotics since they can be included in food, feed or used as tablets. These dietary fibers differ in chemical and physico-chemical properties from fibers of terrestrial origin, their content is higher than in most fruits and vegetables and ranges from 33 to 50 g per 100 g of algae [123]. Some of the algae polysaccharides have already taken as dietary prebiotics and can be used in the complex treatment of IBD. These are low molecular weight polysaccharides LMW-PS, AGAROS (oligosaccharides—derivatives of agarose), xylooligosaccharides, galactose-oligosaccharides (GOS), neoagaro-oligosaccharides (NAOS), oligosaccharides-derivatives of alginate (ALGOS, arabicanic) [124]. Other polysaccharides are studied in experiments [126,127]. The data regarding the possibility of fermentation of fucoidan by intestinal microflora are contradictory, and today they cannot be considered final. Despite the evidence for the prebiotic activity of sulfated polysaccharides in vitro, there is an obvious delay in taking them as prebiotics. This circumstance may be associated with limited in vivo studies and insufficient clinical trials of the prebiotic potential of brown algae polysaccharides [125]. Enzymatic cleavage of α-1→3-bonds in the fucoidan molecule with the formation of sulfated oligosaccharides and fucose is carried out by fucoidanases, mainly isolated from marine fungi and bacteria and mollusks [128]. In this regard, some researchers believe that fucoidan is not fermented by intestinal microflora [126]. At the same time, other authors prove the prebiotic properties of sulfated polysaccharides of brown algae. Lynch et al. [129] found an increase in the total concentration of fatty acids in the proximal and distal parts of the colon of pigs, in the diet of which was added purified fucoidan. Stimulation of growth and the accumulation of biomass of bifidobacteria during their cultivation on a nutrient medium enriched with fucoidan from *Fucus evanescens* are shown by Kuznetsova et al. [130]. The prebiotic properties of the polysaccharide were confirmed in vivo in a model of drug dysbacteriosis in mice after a month-long treatment with a fermented milk drink with *B. bifidum* enriched with polysaccharides from brown alga *F. evanescens* [131]. An increase in *Lactobacillus* and *Ruminococcaceae* in the microbiota of the cecum of mice injected with fucoidan from *Ascophyllum nodosum* and *L. japonica* was noted in Reference [132]. Encouraging results suggesting the possibility of degradation of fucoidan by intestinal bacteria were obtained by Kong et al. [133]. The possibility of transforming fucoidan in the human body is also confirmed by the results of recent studies on the detection of fucoidan in peripheral blood after oral administration of the polysaccharide [134].

The above data show that algae polysaccharides have great potential for use as prebiotics in IBD and can realize health effects, including regulation of the composition and functions of microbiota, lowering the pH in the colon lumen and preventing intestinal colonization by pathogens, and reducing the production of reactive oxygen species providing energy sources for colonocytes and activating free fatty acid receptors. However, further studies of the prebiotic properties of oligo- and polysaccharides from algae are needed to obtain more complete information about their intestinal benefits, including in IBD.

## 5. Drug Delivery Systems based on Seaweed Polysaccharides

The problem of developing new technologies for the delivery of drugs with desired properties to therapeutic targets remains relevant at present [135]. The delivery of orally administered drugs to the colon is also highly desirable for the treatment of IBD [75]. However, targeting oral drugs to the colon, which is located at the distal end of the gastrointestinal tract, is difficult due to physiological problems, biochemical and environmental barriers, including those associated with mucus and epithelium [136]. Various polymer architectures (linear, branched main chains) and polymer combinations are proposed as carrier systems [137]. Marine polysaccharides have great potential for drug delivery systems because they are abundant, cheap, biocompatible and biodegradable, have a wide range of biological activity. Fucoidan deserves special attention among them. Sulfated polysaccharides have been shown to be endowed with specific features that enable this targeting ability. Some fragments of polysulfated fucoidan chains can recognize cell surface receptors, including those specific for mannose, fucose, galactose and N-acetylglucosamine residues, as well as TLR4, CD14, scavenger receptors and the mitogen-activated protein kinase receptor, and modulate the effects of signaling molecules in cells. The pleiotropic effect of sulfated polysaccharides of brown algae is due to their structural features. Some fragments of polysulfated fucoidan chains can recognize cell surface receptors, including those specific for mannose, fucose, galactose and N-acetylglucosamine residues, as well as TLR4, CD14, scavenger receptors and the mitogen-activated protein kinase receptor, and modulate the effects of signaling molecules in cells [30,137]. Therefore, sulfated polysaccharides can be used as specific recognition signals for targeting cells of the immune system and promote the accumulation of the drug in the inflamed intestine. The application of polysaccharides in pharmaceutical formulations includes their use in the manufacture of solid monolithic matrix systems, implants, films, beads, microparticles, nanoparticles, inhalable and injectable systems, as well as hydrogel formulations [30].

Recent preclinical studies have shown that nanoparticle-based drug delivery systems using algal polysaccharides can be promising tools with potentially effective results in the treatment of IBD [75]. Carrageenan and fucoidan in combination with chitosan are most often mentioned as a matrix material in the development of nanoparticles [138,139]. Interactions between the amino group of chitosan and the sulfate group of polysaccharides allow the formation of nanoparticles and limit drug release [138]. Moreover, the pH-responsive profile of fucoidan–chitosan nanoparticles prevents degradation under acidic conditions of the gastrointestinal tract and allows drug absorption in the intestine. For this reason, fucoidan and chitosan nanoparticles have been extensively studied for oral delivery of active pharmaceutical ingredients [30,75,139].

Alginate-chitosan nanoparticles cross-linked by zinc ions and intended for the joint delivery of zinc and 5-aminosalicylic acid showed an excellent therapeutic effect in reducing inflammation in rats with induced colitis [140].

Wu et al. [76] investigated the effect of the berberine-loaded chitosan/fucoidan nanoparticles in protecting intestinal tight-junction barrier function against nitric oxide and inflammatory cytokines released from LPS-stimulated macrophage in a Caco-2 cells/RAW264.7 cell co-culture system. Berberine release from the nanoparticles had a fast release in simulated intestinal fluid (SIF, pH 7.4), while the release was slow in simulated gastric fluid (SGF, pH 2.0). Nanoparticles provided a decrease in TJ disturbance induced by LPS and helped restore barrier function in inflamed and damaged intestinal epithelium.

Lee et al. [75] also used co-cultivation of Caco-2 and RAW264.7 to evaluate soluble chitosan/fucoidan nanoparticles with egg shell protein membranes for treating defective intestinal epithelial cells. Nanoparticles effectively reduced NO and inhibited the production of TNF-α and IL-6 and protected epithelial cells from destruction. Furthermore, fucoidan can help the nanoparticles to target intestinal epithelial cells due to the fucose receptor on the epithelial cells. The authors consider chitosan and fucoidan nanoparticles to be potential carriers for drug delivery that reduce inflammation of the intestinal epithelium

Zhu et al. [141] constructed a biologically active complex—selenium nanoparticles coated with a polysaccharide from the green alga *Ulva lactuca*, possessing low toxicity and high anti-inflammatory activity. The complex had low toxicity and high anti-inflammatory activity. Supplementation with ULP-SeNPs resulted in a significant protective effect on DSS-induced acute colitis in mice including mitigation of body weight loss, and colonic inflammatory damage. ULP-SeNPs ameliorated macrophage infiltration as evidenced by decreased CD68 levels in colon tissue sections. The plasma levels of TNFα and IL-6, COX-2, and iNOS in these animals decreased compared to control animals not receiving the complex. Mechanistically, ULP-SeNPs inhibited the activation of macrophages by suppressing the nuclear translocation of NF-κB, which drives the transcription of these pro-inflammatory cytokines.

Wang et al. [142] used sodium alginate in combination with chitosan to create icariin-loaded microspheres. This drug is poorly soluble in water and has low bioavailability. The microspheres loaded with icariin could not only reduce the colonic injury by decreasing the colon mucosa damage index in rats but also reduce the inflammatory response by reducing the production and gene expression of inflammatory mediators and cytokines in colonic mucosa. All the results indicate that targeted microspheres loaded with icariin could exert the colon-protective effects through reducing the inflammatory response, which would be developed as a potential drug controlled release system for the treatment of ulcerative colitis.

Enhanced degradation of chitosan particles by enterobacteria of the colon is also a useful strategy for targeting drug release to the distal intestine. Crcarevska et al. [143] orally administered rats with colitis-induced microparticles of chitosan-Ca alginate loaded with budesonide coated with Eudragit S-100. The results showed a significant reduction in the severity of colitis after the application of particles coated with Eudragit S-100, compared with particles without coating, and with one budesonide.

Chang et al. [144] used amphiphilic thiolated sodium alginate for site-specific drug delivery in inflammatory bowel diseases. Nanospheres were synthesized by self-assembly of amphiphilic thiolated sodium alginate in deionized water, followed by crosslinking of a disulfide bond. The drug release in pH 6.0 buffer with GSH from drug-loaded nanospheres exhibited a marked increase, indicating that the nanospheres may be used for colon-specific drug delivery.

These studies show the potential of IBD treatment strategies using polysaccharide-based seaweed drug delivery systems. Nevertheless, for the successful application of this strategy in the treatment of IBD, further comprehensive studies of the patterns of accumulation of substances in the intestinal tissues are necessary.

## 6. Conclusions

Inflammatory bowel disease is a serious health concern among western societies worldwide. Current therapeutic strategies that use anti-inflammatory drugs, immunosuppressants, and biological treatments are often ineffective and have adverse health effects. In this context, the use of natural products has attracted widespread attention. Seaweed is a valuable source of structurally diverse bioactive compounds. The cell walls of algae are rich in polysaccharides with a wide range of biological activity. The presented materials indicate that seaweed extracts and polysaccharides are effective candidates for the development of drugs, biological food additives, and functional nutrition products for the treatment and prevention of IBD. The high therapeutic effectiveness of the extracts is due to the harmonious combination and interaction of a wide range of biologically active substances (polysaccharides, flavonoids, pectins, etc.) in their composition, which helps to enhance the pharmacological properties of each incoming ingredient and corresponds to the multivalence of the disease pathogenesis. The pleiotropic effect of sulfated polysaccharides of brown algae, due to their structural features, provides their ability to exert an effect on therapeutic IBD targets, including proinflammatory cytokines, adhesion molecules, nuclear factor NF-kB, and reactive oxygen and nitrogen forms. The prebiotic properties of oligo- and polysaccharides from seaweed are also an attractive alternative for both prophylactic and therapeutic intervention in IBD. Finally, algal polysaccharides have great potential in the development of drug delivery systems since the physicochemical properties of these carbohydrates provide the possibility of interaction with other compounds, whether drugs, proteins or other polymers.

However, currently, the majority of studies regarding the effectiveness of algal polysaccharides in IBD have been carried out in experiments on animals or ex vivo using material from patients suffering from these diseases. This circumstance is associated with difficulties in obtaining standardized drugs, which could be the basis for the creation of dosage forms. Undoubtedly, the use of algal polysaccharides in medicine will expand every year due to the further development of science and the expansion of the possibilities of obtaining standardized preparations based on these compounds. The lack of theoretical knowledge about the influence of specific structural parameters of algal polysaccharides on their biological properties is also a limiting factor. In this regard, the molecular architecture of polysaccharides and the role of structural elements in the manifestation of their biological properties are still the subject of active study and are a logical step towards the creation of drugs based on them.

We hope that further study of the relationship between the effect of polysaccharides from different types of algae, with different structure and molecular weights on immune and epithelial cells, intestinal microorganisms will contribute to a deeper understanding of their mechanisms and will help in the development of drugs, dietary supplements, functional foods for the treatment of patients with IBD.
